# The Impact of Preoperative Eating Habits on Weight Loss After Metabolic Bariatric Surgery

**DOI:** 10.1007/s11695-025-07766-z

**Published:** 2025-03-05

**Authors:** Muhammer Ergenç, Tevfik Kıvılcım Uprak, Hale Feratoğlu, Ömer Günal

**Affiliations:** Department of General Surgery, Marmara University School of Medicine, Istanbul, Turkey

**Keywords:** Metabolic and bariatric surgery, Eating behavior, Weight loss, Body mass index, Obesity, Binge eating disorder

## Abstract

**Background:**

Eating disorders and disordered eating habits are frequently identified among metabolic and bariatric surgery (MBS) patients. However, how these factors may affect postsurgical outcomes has not been adequately addressed. The aim of this study was to investigate the associations between patients’ eating habits and optimal clinical response after MBS.

**Methods:**

The study analyzed data from patients who underwent MBS at Marmara University Hospital between 2015 and 2023. Patient demographics, body mass index, obesity-associated medical problems, follow-up periods, surgical procedures (laparoscopic sleeve gastrectomy-SG, laparoscopic Roux-en-Y gastric bypass-RYGB), and eating habits (binge eating, carbohydrate craving, night eating, and sweet eating) were analyzed. Patients were divided into two groups: the optimal clinical response group (%TWL ≥ 20) and the suboptimal clinical response group (%TWL < 20) after surgery, and the groups were compared.

**Results:**

A total of 426 patients, including 197 patients who underwent SG and 229 patients who underwent RYGB, were included. The mean age of all patients was 40.9 ± 10.7 years. During the preoperative period, binge eating, carbohydrate craving, night eating, and sweet eating habits were detected in 55.9%, 67.6%, 47.7%, and 60.6% of the patients, respectively. The mean follow-up period was 24 months (1–60 months). Eighty percent of the patients achieved an optimal clinical response. The preoperative eating habits and %TWL values of the patients were analyzed. There was no statistically significant effect of eating habits on the optimal clinical response in all patients or in the SG vs RYGB groups.

**Conclusions:**

This study investigated the effect of preoperative disordered eating habits on weight loss after MBS and revealed no significant difference between those with these habits and those without these habits. It is difficult to predict weight loss after MBS on the basis of preoperative eating behavior. However, further studies are needed to evaluate this factor in combination with other factors before or after surgery.

## Introduction

Metabolic and bariatric surgery (MBS) is the most effective treatment for severe obesity, but it does not guarantee long-term stability or sufficient weight loss. Approximately 20 to 30% of patients fail to lose enough weight or gain it back significantly, leading to the recurrence of obesity-related medical problems and a decline in quality of life. Optimizing weight reduction in patients with obesity after surgery is challenging because various factors affect postoperative weight loss and potential weight regain, with debate over the main risk factors [1–7].

Psychiatric disorders, such as anxiety, depression, and binge eating disorders, significantly impact weight loss in patients who undergo MBS. A multidisciplinary team approach is recommended for surgical treatment, and preoperative psychological assessments are crucial. Weight gain may be linked to adherence to dietary guidelines, disordered eating habits, and emotional eating, which can impact weight control [1–5].

Eating disorders, particularly binge eating disorder, are prevalent among MBS patients, with studies indicating that these disorders can predict weight loss postsurgery. However, the impact of these eating disorders on postsurgical outcomes remains unclear. Further research is needed to understand how these eating disorders can be used as indicators for surgical evaluation [3, 6, 8–11].

Carbohydrate craving, sweat eating, and night eating may sometimes appear as an eating habit but not a disorder. Few tests and scales are being used to detect these habits and determine whether they become disorders. We intend to determine whether there is any relationship between these eating behaviors or habits and weight loss after MBS. Therefore, the authors developed eating habit evaluation criteria.

The aim of this study was to investigate the associations between patients’ eating habits and optimal clinical response after MBS.

## Materials and Methods

This was a single-center retrospective study. An analysis was performed on the data of patients who were followed up in the general surgery outpatient clinic following MBS for severe obesity at Marmara University Pendik Training and Research Hospital between February 2015 and October 2023. The Marmara University School of Medicine Clinical Research Ethics Committee approved this investigation (number: 09.2023.1634).

The following data, which were obtained during the preoperative period and postoperative outpatient control, were analyzed: demographic characteristics, height, weight, body mass index (BMI), obesity-associated and other medical problems (diabetes mellitus, hypertension, hypothyroidism, coronary artery disease, dyslipidemia, obstructive sleep apnea, other diseases, e.g., rheumatoid arthritis, chronic venous disease, polycystic ovary syndrome, asthma, psoriasis, migraine, glaucoma), smoking status, follow-up period, surgical procedures—laparoscopic sleeve gastrectomy (SG) and laparoscopic Roux-en-Y gastric bypass (RYGB)—and eating habits (binge eating, carbohydrate craving, night eating, and sweet eating) which were assessed during these examinations.

All the data were collected at the time of each outpatient visit and stored in the hospital’s electronic health record system. The outcome measurements of the patients were followed up at regular intervals for up to sixty months. All the data were prospectively collected and retrospectively reviewed.

The change in BMI (ΔBMI) and percentage of total weight loss (% TWL) were computed on the basis of each patient’s weight at the final follow-up [8, 9].

We defined good responders to MBS as those with a %TWL ≥ 20. Patients were divided into two groups: the optimal clinical response group (%TWL ≥ 20) and the suboptimal clinical response group (%TWL < 20) after surgery, and the groups were compared [12].

### Inclusion Criteria

Patients aged ≥ 18 years with a BMI ≥ 40 kg/m^2^ or BMI ≥ 35 kg/m^2^ and obesity-associated medical problems and patients without active psychiatric disorders (including eating disorders) at psychiatric consultations who underwent primary MBS (SG or RYGB) were included.

### Exclusion Criteria

Patients with missing follow-up data, patients who underwent operations other than SG and RYGB and whose dietary habits were not questioned, patients with drug use or substance abuse affecting weight or appetite, and patients with untreated mental illness were excluded from the study.

Although binge eating disorder (BED) is present in a significant proportion of the bariatric population, the ratio of patients with subclinical binge eating behavior is greater [13]. Patients who were diagnosed with eating disorders according to the scales used in the preoperative psychiatric evaluation and who were not suitable for surgery were excluded.

### Binge Eating

In previous studies, binge eating was defined as follows: According to the DSM-IV, a patient was classified as having binge eating if they agreed to currently experience (for example, past six months) the following criteria: eating more food in a shorter length of time than most people would be in a comparable situation and losing control over their eating during the episode [3, 8, 14].

We asked the questions in Table 1 to the patients who applied for surgery in the general surgery outpatient clinic, where we followed patients with obesity. We evaluated patients’ binge eating habits according to Table 1.

**Table 1:** Eating habits assessment criteria

Eating habits	Assessment	Questions
Binge eating	In our general surgery outpatient clinic, where we follow obesity patients, we asked the following three questions to the patients who applied for surgery. If the patient answered yes to at least one of them, we determined that the patient had a binge eating habit	1. Do you continue to eat even though you are full at any meal? 2. What is your favorite food (e.g., doner kebap, meatballs, hamburgers), and if this favorite food was served to you after a full meal, would you eat it even though you were full? 3. Do you repeat this eating habit more than once a week?
Carbohydrate craving	We asked the following two questions to patients. If one of these conditions is met at least once a week, we determined that the patient had a carbohydrate craving habit	1. When you are hungry, what do you prefer if offered fresh vegetables and bakery products to satisfy your hunger? (We accept the answer “bakery” as positive) 2. How many of your daily meals do you consume pastries? (We accept more than thirty percent positive.)
Night eating	We asked the following two questions to patients. If one of these two conditions is met at least once a week, we determined that the patient had a night eating habit	1. Do you eat less than two hours before bedtime sleep? 2. Do you wake up at night to eat?
Sweet eating	We asked the following two questions to patients. If one of these two conditions occurs more than twice a week, we determined that the patient had a sweet eating habit	1. Do you primarily prefer sweet foods or other foods to satisfy your hunger? 2. Do you consume sweet biscuits, jam, ice cream, sugary drinks, candy, confectionery, wafers, chocolate, sweets, cakes, etc., during visual activities such as watching television or reading books?

### Carbohydrate Craving

The Food Craving Inventory (FCI), the main measure of interest, was utilized to look at both food cravings and food consumption. On a 5-point Likert scale from 1 (never) to 5, respondents were asked to rate how frequently they had wanted each of 28 food items over the previous 30 days (always). The participants were also questioned about how frequently they consumed a specific dish that they might have had a craving for. In addition to scores for four subscales reflecting mean ratings for high-sugar, high-fat, high-carb, and fast foods, an overall food yearing score (mean of all 28 items) was also computed. Higher scores imply greater food cravings [15–17].

We evaluated patients’ carbohydrate craving habits according to Table 1.

### Night Eating

Three or more times a week, a person who ingested at least half of their daily calories after 7 PM, had trouble falling asleep, and claimed not to be hungry at breakfast was classified as a “night eater” [14, 18, 19].

We evaluated patients’ night eating habits according to Table 1.

### Sweet Eating

A person who consumes at least 50% simple carbs is referred to as a “sweet-eater” [14, 17, 19].

We evaluated patients’ sweet eating habits according to Table 1.

### Statistical Analysis

The IBM Corporation-developed Statistical Package for the Social Sciences (SPSS, Version 24 for Mac) was used to perform the statistical analyses. Data that were not normally distributed were represented by median values, while normally distributed data were shown as the mean ± standard deviation. The category data were compared using the chi-square test. The Student’s *t*-test was employed to compare parametric data, whereas the Mann–Whitney *U* test was utilized to examine nonparametric data. Statistical significance was defined as a two-sided *p*-value less than 0.05 with a 95% confidence range.

## Results

The data of 491 patients who were followed up in the hospital’s MBS outpatient clinic between February 2015 and October 2023 were analyzed for the study. Five patients with missing preoperative data, 15 patients with missing postoperative data, nine patients with missing eating habits, seven patients treated nonsurgically, 15 patients who underwent transit bypass surgery, 14 patients who underwent revision surgery were excluded, and 426 patients were included in the study.

The mean age of all patients was 40.9 ± 10.7 years. During the preoperative period, binge eating, carbohydrate craving, night eating, and sweet eating habits were detected in 55.9%, 67.6%, 47.7%, and 60.6% of the patients, respectively. The mean follow-up period was 24 months (range: 1–60 months). Eighty percent of the patients achieved an optimal clinical response (Table 2).

**Table 2:** Patient characteristics according to surgical procedure

Variables	All patients *n* = 426	SG *n* = 197	RYGB *n* = 229	*p* value
Age (years, mean ± SD)	40.9 ± 10.7	40.3 ± 11.1	41.5 ± 10.44	0.25
Sex (female – *n*, %)	352 (82.6)	162 (82.2)	190 (83)	0.84
Diabetes mellitus (*n*, %)	137 (32.2)	55 (27.9)	82 (25.8)	0.08
Hypertension (*n*, %)	101 (23.7)	41 (20.8)	60 (26.2)	0.19
Hypothyroidism (*n*, %)	53 (12.4)	21 (10.7)	32 (14)	0.37
Coronary artery disease (*n*, %)	6 (1.4)	3 (1.5)	3 (1.3)	0.58
Dyslipidemia (*n*, %)	16 (3.8)	5 (2.5)	11 (4.8)	0.33
Obstructive sleep apnea (*n*, %)	45 (10.6)	12 (6.1)	33 (14.4)	**0.009**
Other disease (*n*, %)	85 (20)	41 (20.8)	44 (19.2)	0.68
Smoking (*n*, %)	101 (23.7)	41 (20.8)	60 (26.2)	0.19
Preoperative BMI (kg/m^2^, mean ± SD)	48.3 ± 7.4	48.7 ± 8.1	47.8 ± 6.7	0.27
Binge eating (*n*, %)	238 (55.9)	109 (55.3)	129 (56.3)	0.83
Carbohydrate craving (*n*, %)	288 (67.6)	130 (66)	158 (69)	0.50
Night eating (*n*, %)	203 (47.7)	93 (47.2)	110 (48)	0.86
Sweet eating (*n*, %)	258 (60.6)	127 (64.5)	131 (57.2)	0.12
Postoperative BMI (kg/m^2^, mean ± SD)	31.8 ± 6.1	31.7 ± 6.4	31.8 ± 5.8	0.81
Follow-up period (months, mean ± SD) (range: 1–60)	24.1 ± 16.4	24.9 ± 18.4	23.4 ± 14.4	0.34
Change in BMI (kg/m^2^, mean ± SD)	16.5 ± 7.1	16.9 ± 7.65	16.1 ± 6.6	0.17
%TWL (mean ± SD)	33.7 ± 11.7	34.1 ± 12.6	33.4 ± 10.8	0.53
Optimal weight loss (%TWL ≥ 20 – *n*, %)	378 (88.7)	172 (87.3)	206 (90)	0.38

*SG*, laparoscopic sleeve gastrectomy; *RYGB*, laparoscopic Roux-en-Y gastric bypass; *SD*, standard deviation; *BMI*, body mass index; *%TWL*, %total weight loss

We compared 197 patients who underwent SG with 229 patients who underwent RYGB. There was no significant difference between the groups in terms of obesity-associated medical problems except obstructive sleep apnea. The mean preoperative BMI and percentage of preoperative eating habits were similar between the SG and RYGB groups. There was no significant difference between the two groups in terms of the change in BMI (ΔBMI) or the percentage of total weight loss (% TWL) **(**Table 2**)**.

**Fig. 1:**
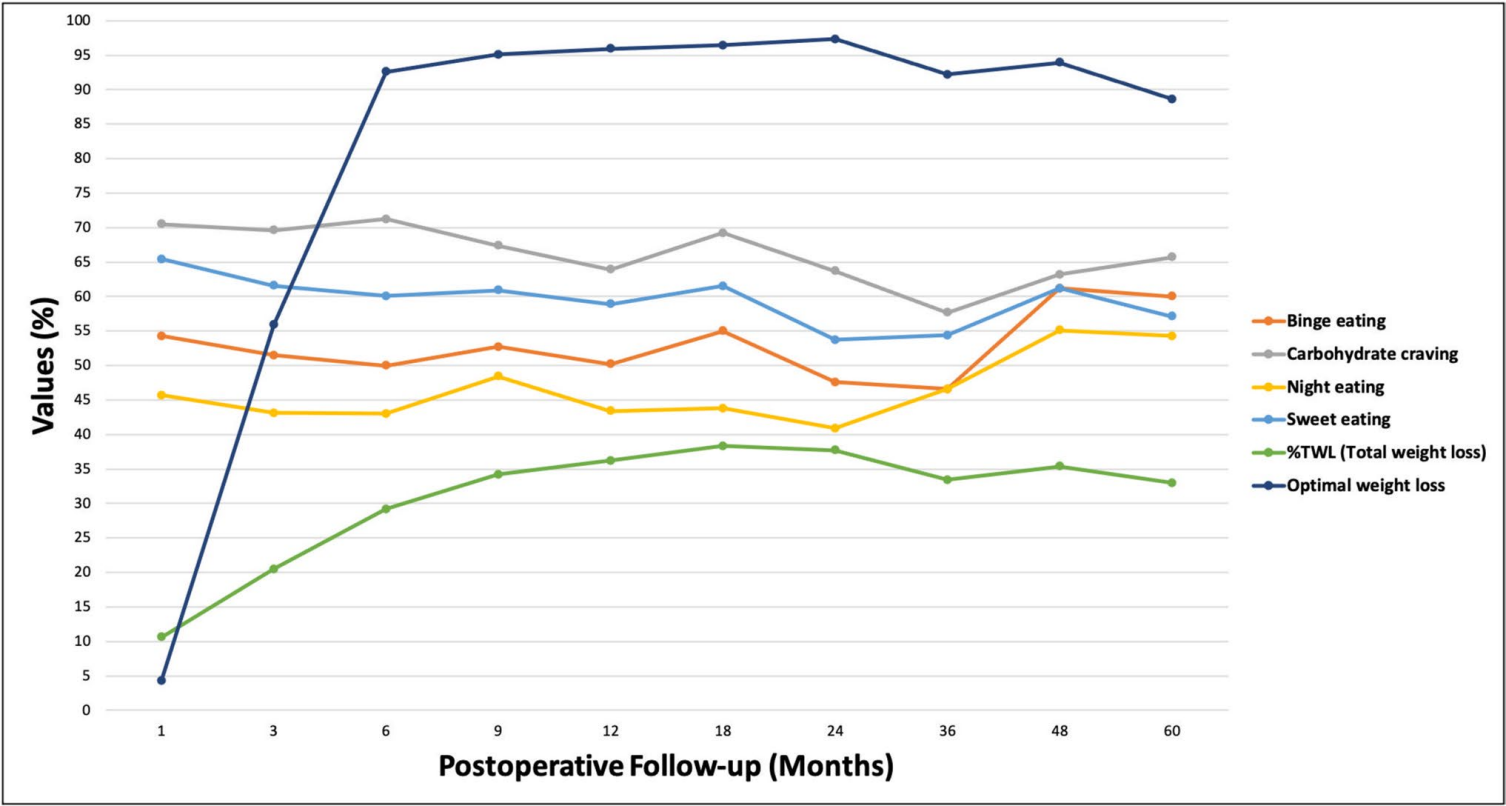
Weight loss curves and eating habits over time

The number of patients attending regular follow-ups decreased as the follow-up period was prolonged. The incidence of eating habits generally declined until the 3rd year of follow-up and subsequently increased. The percentage of patients with an optimal clinical response was high in the first two years and decreased in the 3rd, 4th, and 5th years **(**Table 3**)**. The relationships between weight loss curves and eating habits over time are shown in Fig. 1.

**Table 3:** Postoperative follow-up data on weight loss parameters and eating habits

Variables	All patients – Preoperative *n* = 426	1st month *n* = 234	3rd month *n* = 227	6th month *n* = 216	9th month *n* = 184	12th month *n* = 219	18th month *n* = 169	24th month *n* = 149	36th month *n* = 90	48th month *n* = 49	60th month *n* = 35
Loss to follow-up (*n*, % of 426 patients)		192 (45.1)	199 (46.7)	210 (49.3)	242 (56.8)	207 (48.6)	257 (60.3)	277 (65)	336 (78.9)	377 (88.5)	391 (91.8)
Binge eating (*n*, %)	238 (55.9)	127 (54.3)	117 (51.5)	108 (50)	97 (52.7)	110 (50.2)	93 (55)	71 (47.6)	42 (46.6)	30 (61.2)	21 (60)
Carbohydrate craving (*n*, %)	288 (67.6)	165 (70.5)	158 (69.6)	154 (71.2)	124 (67.4)	140 (63.9)	117 (69.2)	95 (63.7)	52 (57.7)	31 (63.2)	23 (65.7)
Night eating (*n*, %)	203 (47.7)	107 (45.7)	98 (43.1)	93 (43)	89 (48.4)	95 (43.4)	74 (43.8)	61 (40.9)	42 (46.6)	27 (55.1)	19 (54.3)
Sweet eating (*n*, %)	258 (60.6)	153 (65.4)	140 (61.6)	130 (60.1)	112 (60.9)	129 (58.9)	104 (61.5)	80 (53.7)	49 (54.4)	30 (61.2)	20 (57.1)
Postoperative BMI (kg/m^2^, mean ± SD)		42.2 ± 6.3	37.6 ± 5.7	33.8 ± 5.5	31.4 ± 4.8	30.7 ± 5	30 ± 4.9	30.2 ± 5	32.3 ± 5.6	32.7 ± 5.9	33.5 ± 6.9
Change in BMI (kg/m^2^, mean ± SD)		4.9 ± 2.8	9.7 ± 3.1	14.1 ± 4	16.5 ± 5	17.8 ± 5.7	18.9 ± 5.8	18.8 ± 6.9	16.8 ± 7	18.8 ± 7	17 ± 7.6
%TWL (mean ± SD)		10.6 ± 5.8	20.5 ± 5.7	29.2 ± 6.7	34.2 ± 8	36.2 ± 8.8	38.3 ± 8.7	37.7 ± 9.7	33.4 ± 10.4	35.4 ± 10.3	33 ± 12
Optimal weight loss (%TWL ≥ 20 – *n*, %)		10 (4.3)	127 (55.9)	200 (92.6)	175 (95.1)	210 (95.9)	163 (96.4)	145 (97.3)	83 (92.2)	46 (93.9)	31 (88.6)

Preoperative binge eating, carbohydrate craving, night eating, and sweet eating habits and the % TWL values of the patients were analyzed. There was no statistically significant effect of eating habits on the optimal clinical response in all patients or in the SG vs RYGB groups **(**Table 4**)**.

**Table 4:** Relationships between weight loss and eating habits

Surgical procedure		Preoperative binge eating				Preoperative carbohydrate craving			
		Absent (*n*, %)	Present (*n*, %)	Total (*n*, %)	*p* value	Absent (*n*, %)	Present (*n*, %)	Total (*n*, %)	*p* value
SG	%TWL < 20	9 (10.2)	16 (14.7)	25 (12.7)	0.351	9 (13.4)	16 (12.3)	25 (12.7)	0.822
	%TWL ≥ 20	79 (89.8)	93 (85.3)	172 (87.3)		58 (86.6)	114 (87.7)	172 (87.3)	
	Total	88 (100)	109 (100)	197 (100)		67 (100)	130 (100)	197 (100)	
RYGB	%TWL < 20	12 (12)	11 (8.5)	23 (10)	0.386	5 (7.0)	18 (11.4)	23 (10.0)	0.311
	%TWL ≥ 20	88 (88)	118 (91.5)	206 (90)		66 (93)	140 (88.6)	206 (90)	
	Total	100 (100)	129 (100)	229 (100)		71 (100)	158 (100)	229 (100)	
All procedures	%TWL < 20	21 (11.2)	27 (11.3)	48 (11.3)	0.955	14 (10.1)	34 (11.8)	48 (11.3)	0.612
	%TWL ≥ 20	167 (88.8)	211 (88.7)	378 (88.7)		124 (89.9)	254 (88.2)	378 (88.7)	
	Total	188 (100)	238 (100)	426 (100)		138 (100)	288 (100)	426 (100)	

Preoperative night eating				Preoperative sweet eating			
Absent (*n*, %)	Present (*n*, %)	Total (*n*, %)	*p* value	Absent (*n*, %)	Present (*n*, %)	Total (*n*, %)	*p* value
11 (10.6)	14 (15.1)	25 (12.7)	0.346	7 (10.0)	18 (14.2)	25 (12.7)	0.400
93 (89.4)	79 (84.9)	172 (87.3)		63 (90.0)	109 (85.8)	172 (87.3)	
104 (100)	93 (100)	197 (100)		70 (100)	127 (100)	197 (100)	
13 (10.9)	10 (9.1)	23 (10)	0.645	12 (12.2)	11 (8.4)	23 (10)	0.338
106 (89.1)	100 (90.9)	206 (90)		86 (87.8)	120 (91.6)	206 (90)	
119 (100)	110 (100)	229 (100)		98 (100)	131 (100)	229 (100)	
24 (10.8)	24 (11.8)	48 (11.3)	0.730	19 (11.3)	29 (11.2)	48 (11.3)	0.982
199 (89.2)	179 (88.2)	378 (88.7)		149 (88.7)	229 (88.8)	378 (88.7)	
223 (100)	203 (100)	426 (100)		168 (100)	258 (100)	426 (100)	

*SG*, laparoscopic sleeve gastrectomy; *RYGB*, laparoscopic Roux-en-Y gastric bypass; suboptimal weight loss: %TWL < 20, optimal weight loss: %TWL ≥ 20

## Discussion

Approximately half of patients undergoing MBS have some form of disordered eating behavior. Loss of control eating may be the most common disordered eating behavior, characterized by a subjective sense of loss of control over eating. Anxiety, depression, and binge eating disorders are the most common psychiatric disorders reported in MBS patients. Many studies have shown that a significant proportion of people with obesity experience binge eating episodes [20, 21]. In this research, in line with these findings in the literature, we found at least one of some subclinical eating habits. Binge eating, carbohydrate craving, night eating, and sweet eating habits were detected in 55.9, 67.6, 47.7, and 60.6% of the patients evaluated for MBS in our center.

Many studies have investigated the relationship between binge eating disorder and weight loss, but no common conclusion has been reached. Some studies have reported that binge eating has a negative effect on weight loss, and other studies have shown that binge eating has no effect [20, 22–25]. The effect of binge eating on weight loss outcomes in people undergoing MBS may change the management of this condition before and after the operation and may benefit patients [20]. This study revealed that binge eating is an important health problem in patients with obesity, but it does not have a significant effect on weight loss compared with patients without binge eating.

The prevalence of BED in the bariatric population is reported to be between 10 and 50%, and the rate of subclinical binge eating behavior has been reported to be as high as 80%. Patients who binge eat do not differ from nonbinge eating (NBE) patients in terms of preoperative weight status but are often characterized by greater psychosocial problems and significantly more psychiatric issues. MBS has a positive effect on several aspects of eating behavior, including hunger, disinhibition, restrictive eating, and binge eating, at least in short-term studies. Nevertheless, these improvements may diminish when the initial surgical impact wanes [13]. The binge eating habit rate of 55.9% in this study was similar to findings in the literature.

In a study evaluating the effect of the preoperative psychiatric profile on weight loss outcomes in Roux-en-Y gastric bypass patients, high anxiety scores had a negative effect on long-term weight outcomes, but eating behavior had no significant effect on short-term weight change. When the authors evaluated these results with previous studies, they stated that this was an important limitation in previous studies, as restrictive or malabsorptive bariatric procedures would lead to different changes in eating patterns [2]. In this study, we did not find a significant result in terms of weight loss according to dietary habits in patients who underwent SG, which is only a restrictive procedure, or RYGB, which is a mixed procedure.

A study examining sweet cravings and different types of food cravings and the consumption of craved foods by MBS patients revealed that MBS was associated with significant reductions in overall food cravings and total consumption of craved foods at 3 and 6 months postsurgery. Nevertheless, changes in cravings and consumption in surgery patients were largely unrelated to weight loss [16]. A significant proportion of patients undergoing MBS report problematic eating behaviors [26]. In a study examining the relationships between abnormal eating behaviors such as binge eaters, snack eaters, sweet eaters, and volume eaters and weight loss after SG, eating habits did not affect surgical outcomes. However, eating habits may worsen after SG [27].

Night eating syndrome can be found in up to 20% of MBS patients. Symptoms appear to persist after surgery, but there is no evidence that preoperative night eating syndrome negatively affects postoperative weight loss [28]. The literature suggests that 4–45% of patients may meet the criteria for binge eating disorder, 20–60% for grazing, 2–42% for night eating syndrome, 38–59% for emotional eating, and 17–54% for food addiction. It is important for clinicians to understand the potential difficulties with eating experienced by patients before MBS and their expectations of how their eating and hunger status will change after surgery [29]. A bariatric surgeon’s awareness of problematic eating habits may increase the degree of preoperative and postoperative optimization of these problems and the optimal clinical response to the operation.

This study has several limitations. This was a single-center study, which may affect the generalizability of the results. Additionally, the evaluation of long-term weight loss is limited because patients were lost to follow-up, and MBS procedures other than RYGB and SG were not evaluated in the present study. However, a notable strength of this study is the simultaneous evaluation of multiple eating habits. It is also possible that selection bias occurred, which may have influenced the findings. In future research, it would be beneficial to examine the long-term effects of eating habits on weight loss with a larger sample of participants. A prospective study could be designed to evaluate different methods of surgery on the basis of various eating habits. Additionally, the impact of single versus multiple combinations of eating habits on the outcomes could be analyzed.

## Conclusions

In this study, we investigated the effects of preoperative disordered eating habits such as binge eating, carbohydrate cravings, night eating, and sweet eating on weight loss after SG and RYGB surgery and found no significant differences between those with these habits and those without these habits. It is difficult to predict weight loss after metabolic and bariatric surgery on the basis of preoperative eating behavior. However, further studies are needed to evaluate this factor in combination with other factors before or after surgery.

## Data Availability

The datasets generated during and/or analysed during the current study are available from the corresponding author on reasonable request.

## Declarations

Part of the data included in this manuscript was presented as an oral presentation at the 17th National Endoscopic, Laparoscopic, and Robotic Surgery Congress, held in Antalya, Turkey, between 25 and 29 October 2024, and published in the Congress Abstract Book.
